# Elective neck treatment in sinonasal undifferentiated carcinoma: Systematic review and meta‐analysis

**DOI:** 10.1002/hed.26077

**Published:** 2020-01-10

**Authors:** Muhammad Faisal, Rudolf Seemann, Claudia Lill, Sasan Hamzavi, Arno Wutzl, Boban M. Erovic, Stefan Janik

**Affiliations:** ^1^ Department of Otorhinolaryngology, Institute of Head and Neck Diseases, Evangelical Hospital Vienna Vienna Austria; ^2^ Department of Head and Neck Surgery Shaukat Khanum Memorial Cancer Hospital and Research Centre Lahore Pakistan; ^3^ Department of Otorhinolaryngology, Head and Neck Surgery Medical University Vienna Vienna Austria

**Keywords:** elective neck dissection, elective neck treatment, regional relapse, sinonasal undifferentiated carcinoma, SNUC

## Abstract

Sinonasal undifferentiated carcinomas (SNUCs), being an aggressive malignancy with dismal survival outcome, have given limited consideration regarding management of regional failures. A total of 12 studies, published between 1999 and 2019, met inclusion criteria. We performed a meta‐analysis assessing regional (neck) relapse after elective neck treatment compared to observation in clinically node negative (N0) necks. Clinical data of 255 patients were used for meta‐analysis. Among them, 83.4% of patients presented with T4 tumors and 14.1% had positive neck nodes. Elective neck treatment was applied in 49.5% of analyzed patients. Regional relapses occurred in 3.7% of patients who have undergone elective neck treatment compared to 26.4% in patients who had not. Elective neck treatment significantly reduced the risk of regional recurrence (odds ratio 0.20; 95% confidence interval 0.08‐0.49; *P* = .0004). The meta‐analysis indicates that elective neck treatment could significantly reduce the risk of regional failures in patients with SNUCs.

## 1 INTRODUCTION

Sinonasal undifferentiated carcinoma (SNUC) is a rare, highly aggressive malignancy that lacks clearly defined treatment protocols and concrete stage‐based survival data. Overall SNUC mortality rates are high, with 5‐year survival ranging from 20% to 63% in the literature.^1^ SNUCs are characterized by aggressive tumor behavior with locally advanced diseases and poor outcome due to locoregional and distant recurrences.^2^, ^3^

SNUCs were first described by Frierson and coworkers in 1986 as a new distinctive clinicopathologic entity that must be distinguished from other less, aggressive sinonasal malignancies.^4^ Since that several case series, retrospective studies and reviews have been conducted in order to understand pathophysiology, tumor behavior and to improve treatment concept and consequently outcome.^5^, ^6^, ^7^, ^8^, ^9^, ^10^, ^11^, ^12^ There exists no consensus about the optimal treatment in patients with SNUCs, because of the scarcity of the disease. However, trimodal treatment regimens, including surgery, radiotherapy, and chemotherapy are associated with improved outcome compared to bimodal approaches.^13^, ^14^

Moreover, due to the complex anatomical location of these tumors and the advanced tumor stages^14^ in the majority of cases at time of diagnosis, surgical treatment concepts often requiring combined endoscopic and open craniofacial approaches, have mainly focused on radical tumor resection with clear resection margins and good local control. Consequently, the issue of elective neck treatment, and of elective neck dissection (END) in particular, has been usually neglected and not been addressed in initial therapy.

Recently,^37^ demonstrated initial nodal involvement in 16.7% and 32.7% of SNUCs located within the nasoethemoidal complex or beyond that. T‐classification or tumor size had no significant impact on the risk of nodal involvement. Among patients with cN0 disease who did not receive any neck treatment, 9% developed neck failures compared to the absence of regional recurrences in those patients undergoing END.^15^ The role of END in undifferentiated sinonasal carcinomas have remained controversial, but more than 20% risk of occult nodal metastasis needs guidelines to be reviewed for better regional control.^16^, ^17^, ^18^

However, owing to the rarity of these malignancies, clear recommendations regarding elective neck treatment in SNUCs is still lacking. Therefore, we conducted this meta‐analysis to calculate the risk of initial nodal involvement, to assess the risk of regional failure in patients undergoing elective neck treatment compared to those without, and to subsequently evaluate whether elective neck treatment is justified.

## 2 MATERIALS AND METHODS

### 2.1 Search strategies

We carried out a comprehensive search in PubMed, Cochrane Library, Web of Science, EMBASE, Biomedical Literature Database (CBM), and http://clinicaltrials.gov for articles published between 1999 and 2019. Following key words were used for query: “sinonasal undifferentiated tumor,” “sinonasal tumor,” “SNUC,” “Sinonasal malignant tumors,” “SNUC and neck dissection,” and “sinonasal tumors and neck dissection.”

### 2.2 Inclusion and exclusion criteria

For inclusion into the analysis, studies had to fulfill all of the following inclusion criteria: (a) systematic reviews, retrospective studies, literature reviews including management of primary SNUC; (b) studies published between 1999 and 2019; (c) precise information regarding sample size, nodal involvement at time of presentation, type of neck treatment, subsequent regional recurrence, and risk of nodal involvement.

On the other hand, (a) case reports and series including less than five patients, (b) studies reporting on pathology other than SNUCs (eg, sinonasal endocrine tumors, esthesioneuroblastoma, lymphomas, or squamous cell carcinomas), (c) studies that do not provide data on elective neck treatment (neck dissection or irradiation), and (d) letter to editors, meeting abstracts and editorials were excluded from analysis.

### 2.3 Search findings

**Figure 1:**
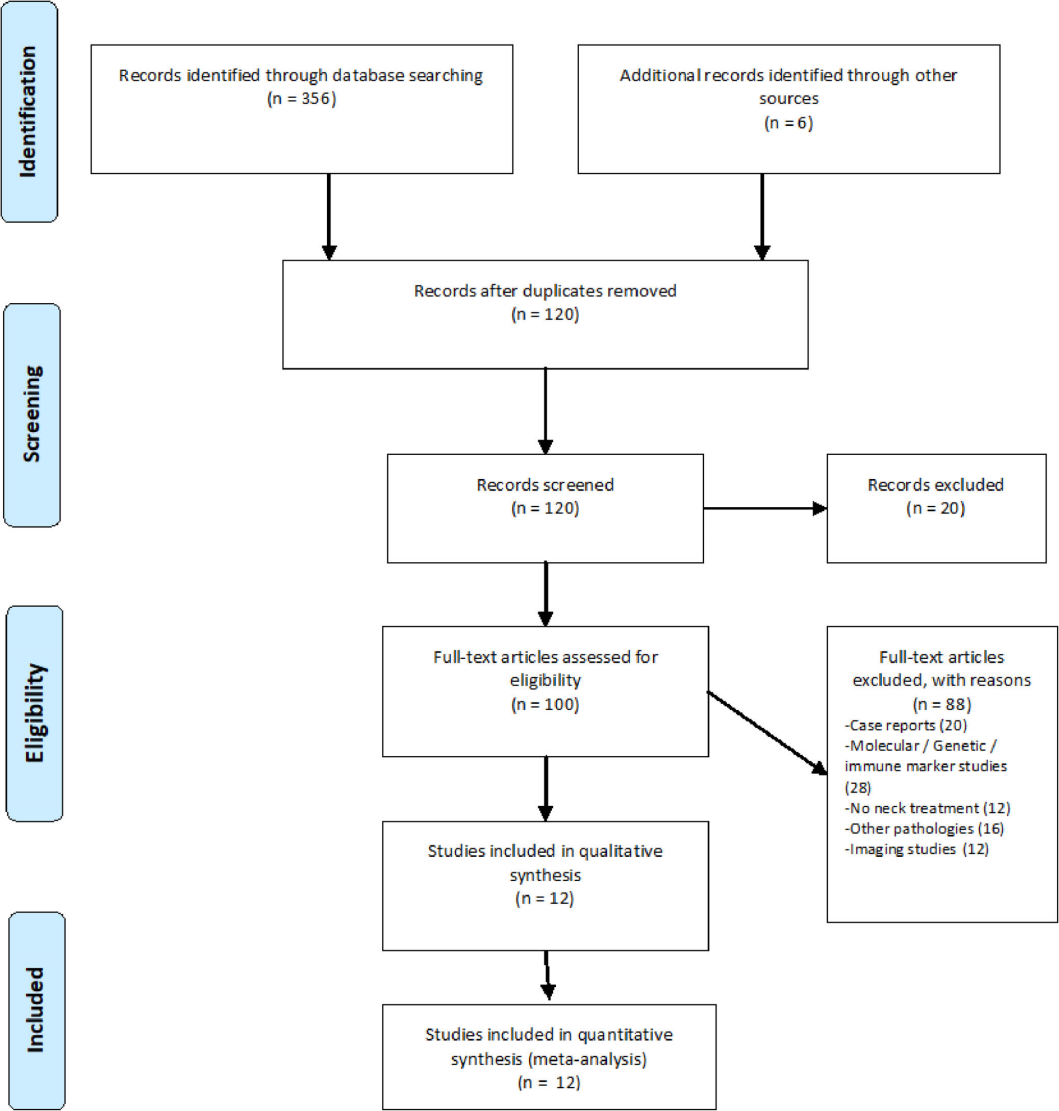
Flow‐diagram. The flow diagram was adapted according to the PRISMA recommendations [Color figure can be viewed at http://wileyonlinelibrary.com]

A total of 356 articles were identified within the database search using the abovementioned keywords. In a first step, duplicates were removed by using Endnote 6. Next, the remaining 120 articles were screened by title and abstract to select for relevant studies. After that step, 37 studies remained for further analysis. Papers reporting on immunohistochemical/pathological/molecular or genetic studies, nonsurgical management without addressing neck and case reports or series with less than five cases were excluded. The final selection of the relevant studies involved an extensive screening of the selected articles by going through the abstracts and the full text studies in doubt. After full‐text revision, 12 articles,^10^, ^13^, ^19^, ^20^, ^21^, ^22^, ^23^, ^24^, ^25^, ^26^, ^27^, ^28^ all published in English with 255 participants were selected for final evaluation. Eight studies^8^ were conducted in the USA, while one study was performed in Switzerland, Spain, India, and France, respectively. We followed the PRISMA guidelines, and the study selection procedure was illustrated by the PRISMA flow diagram (Figure 1).

### 2.4 Data extraction

The cumulative data gathered detailed information on gender distribution, treatment modalities, locoregional control, and follow up. Information was extracted from these cohorts regarding nodal involvement at presentation, treatment for nodal disease and regional recurrences.

The cumulative analysis was performed including modalities used for treatment, nodal involvement at presentation or subsequent recurrence. The estimated risk of nodal involvement was calculated by combining nodal involvement at presentation and subsequent recurrence in neck whether treated with irradiation/selective neck dissection or no treatment at all.

### 2.5 Quality and risk of bias assessment

The quality and risk of bias of all included studies were assessed independently by two authors (M.F., S.J.) based on the A Cochrane Risk of Bias Assessment Tool for Non‐Randomized Studies of Interventions (ACROBAT‐NRSI) criteria (Table 3). Following issues were evaluated: selection of population (a), assessment of exposure (b), outcome of interest (c), match with prognostic variables (d), assessment of prognostic factors (e), assessment of outcome (f), adequate follow‐up (g), and co‐intervention between groups (h). Criteria were categorized as “low risk,” “medium risk,” and “high risk” of bias, respectively. In case of any disagreement, rating was resolved by discussion.

### 2.6 Statistical methods

SPSS (version 26; IBM SPSS Inc., Chicago, Illinois) was used for statistical analysis of data. Mainly descriptive analyses were used. Data are indicated as mean or median ± SD within the result section. Unpaired student's *t* test was used to compare normally distributed means of metric variables. The free available software RevMan 5.3 (Cochrane Collaborative, Oxford, England) was used for the meta‐analysis and creation of the forest plot. The odds ratios (ORs) of regional (neck) nodal recurrence and their 95% confidence intervals (CIs) were calculated for each study. Statistical heterogeneity was assessed using the Cochran *Q* statistic (*P* value for heterogeneity) and the *I* ^2^ statistic (total percentage of variation resulting from heterogeneity). In case of significant heterogeneity (*I* ^2^ ≥ 50) the random‐effect model was used, while the fixed‐effect model was used in absence of significant heterogeneity. Herein, we solely applied the fixed‐effect model to obtain OR, HR, 95% CI, and *P*‐value.

## 3 RESULTS

### 3.1 Study cohort

A total of 12 studies, comprising 10 cohort studies and 2 case series, were included for meta‐analysis, which were published between 2004 and 2017. The entire cohort included 255 patients with a median number of 16.5 cases per work. Information regarding sex was not provided by one study (n = 16), therefore our cohort consisted of 149 males (62.3%) and 90 females (37.7%) with a median age of 51.3 ± 4.4 years (range: 42‐57 years; Table 1).

**Table 1:** Characteristics of the included studies

Study	Year of publication	Country	Study type	Study size	Sex (M:F)	Median age (years)
Al‐Mamgani et al	2013	USA	Cohort study	21	11:10	52
Bhasker et al	2017	INDIA	Case series	16	13:3	47
Chen et al	2008	USA	Cohort study	21	14:7	47
Christopherson et al	2014	USA	Cohort study	23	14:9	56
de Bonnecaze et al	2018	F	Cohort study	54	33:21	54
Gamez et al	2016	USA	Cohort study	40	24:16	56
Kim et al	2004	USA	Case series	8	6:2	42
Lopez et al	2015	E	Cohort study	17	9:8	53
Morand et al	2017	CH	Cohort study	11	8:3	51
Revenaugh et al	2011	USA	Cohort study	13	7:6	51
Tanzler et al	2008	USA	Cohort study	15	10:5	57
Yoshida et al	2013	USA	Cohort study	16	n.p.	50

Abbreviations: F, France; E, Spain; CH, Switzerland; n.p., not provided.

[Correction added on 3rd April 2020, after first online publication: “Morand and Irinakis” has been changed to “Morand et al” in column 1.]

### 3.2 Tumor characteristics

At time of diagnosis, information regarding T‐classification was available in 229 patients. Among them, 83.4% (n = 191), 12.7% (n = 29), and 3.9% (n = 9) were T4, T3, and T1‐T2 carcinomas, respectively. Altogether, positive neck nodes (N+) were described in 14.1% of cases (36 out of 255), ranging from 0% to 25% (Table 2).

**Table 2:** Tumor characteristics and oncological outcome

Study	T‐stage		Nodal stage		Locoregional control			Regional failure	Overall survival		Follow up
	n (%)		n (%)		S(C) RT (%)	S (%)	CRT (%)	n (%)	Years	%	Months
Al‐Mamgani et al	T3	6 (29)	N0	19 (90)	25	—	33	2 (9.5)	5	74	38
	T4	15 (71)	N+	2 (10)							
Bhasker et al	T3	1 (6)	N0	13 (81)	50	—	50	1 (6.3)	n.p.		10
	T4	15 (94)	N+	3 (19)							
Chen et al	T3	4 (19)	N0	19 (90)	47	—	41	2 (9.5)	5	43	58
	T4	17 (81)	N+	2 (10)							
Christopherson et al	T3	1 (4)	N0	18 (78)	69	—	50	6 (26)	5	32	36
	T4	22 (96)	N+	5 (22)							
de Bonnecaze et al	T1‐T2	4 (7.6)	N0	40 (76.9)	54	20	70	7 (13)	3	62.4	43
	T3	9 (17.3)	N+	12 (23.1)							
	T4	39 (75)									
Gamez et al	T1‐T2	3 (7.5)	N0	37 (92.5)	51	—	38	1 (2.5)	5	44	82
	T3	5 (12.5)	N+	3 (7.5)							
	T4	32 (80)									
Kim et al.	C	7 (87.5)	N0	n.p.	75	—	33	3 (37)	2	75	20
	B	1 (12.5)	N+	n.p.							
Lopez et al	T3	1 (5.9)	N0	15 (88)	78	37	100	3 (17.6)	5	58	36
	T4	16 (94.1)	N+	2 (12)							
Morand et al	T2	1 (9.1)	N0	11 (100)	55	—	51	2 (18)	5	36.4	38
	T3	2 (18.2)	N+	0 (0)							
	T4	8 (72.7)									
Revenaugh et al	T1	1 (8)	N0	12 (92)	85	—	66	1 (14)	2	57	32
	T4	12 (92)	N+	1 (8)							
Tanzler et al	T4	15 (100)	N0	13 (86)	100	—	40	2 (13)	3	67	30
			N+	2 (14)							
Yoshida et al	T3	1 (6)	N0	12 (75)	78	37	18	4 (25)	2	75	14
	T4	15 (94)	N+	4 (25)							

Abbreviations: N+, positive neck nodes; S(C)RT, bimodal or trimodal therapy comprising surgery and chemoradiotherapy or radiotherapy; S, surgery; CRT, chemoradiotherapy; n.p., not provided; C (Kadish Stage C), is defined as tumor extension beyond the sinonasal cavities, into the paranasal sinuses with involvement of the cribriform lamina, orbit, skull‐base, or brain; B (Kadish Stage B), is defined as tumor that involves the nasal cavity and one or more paranasal sinuses.

[Correction added on 3rd April 2020, after first online publication: “Morand and Irinakis” has been changed to “Morand et al” in column 1.]

### 3.3 Locoregional control and outcome

**Figure 2:**
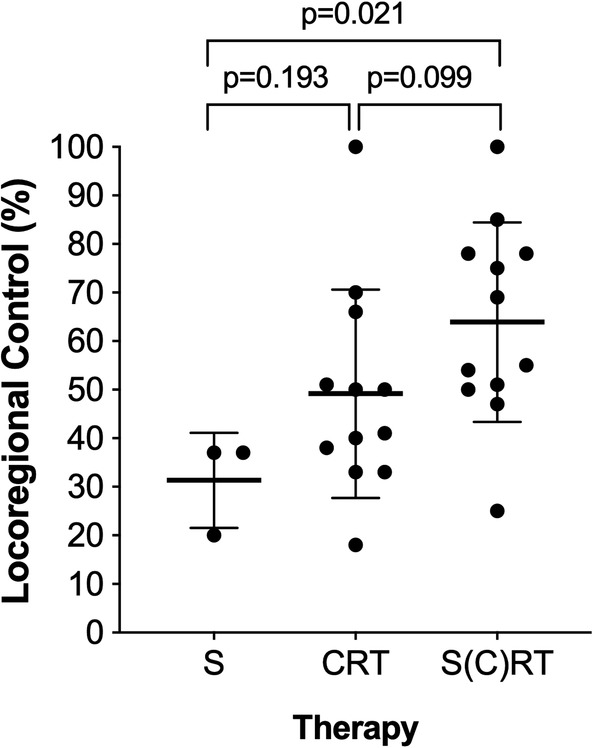
Locoregional control and therapy. The locoregional control (LRC) is illustrated according to treatment regimes. Best LRC was achieved in patients undergoing trimodality therapy (63.9% ± 20.5%), followed by bimodality therapy (49.2% ± 21.5%), and worst LRC was achieved after surgery only (31.3% ± 9.8%). Box‐plots display means and corresponding standard deviations. S, surgery; CRT, chemoradiotherapy; S(C)RT, surgery and (chemo‐) radiotherapy

The median follow‐up was 36.0 ± 19.4 months (range: 10‐82 months). In 9 out of 12 studies (75%), bimodality (chemoradiotherapy—CRT) and trimodality (surgery and [chemo‐] radiotherapy—S[C]RT) treatment regimes were applied. Surgical tumor resection without adjuvant Radiotherapy (RT) or Chemotherapy were reported in three studies. The mean locoregional control (LRC) was 63.9% ± 20.5%, 49.2% ± 21.5%, and 31.3% ± 9.8% for patients undergoing S(C)RT, RCT, or surgery only. LRC was significantly better in patients undergoing trimodality treatment regimes compared to surgery only (*P* = .021), and better but not statistically significant different compared to patients undergoing bimodality therapies (*P* = .099; Figure 2). Regional relapse was reported in 2.5% to 26.1% of cases. The mean 2, 3, and 5 years OS was 69.0%, 64.7%, and 47.9% respectively, which was indicated by the vast majority of studies as one of the main outcome parameters.

### 3.4 Elective neck treatment vs observation in cN0 disease

**Figure 3:**
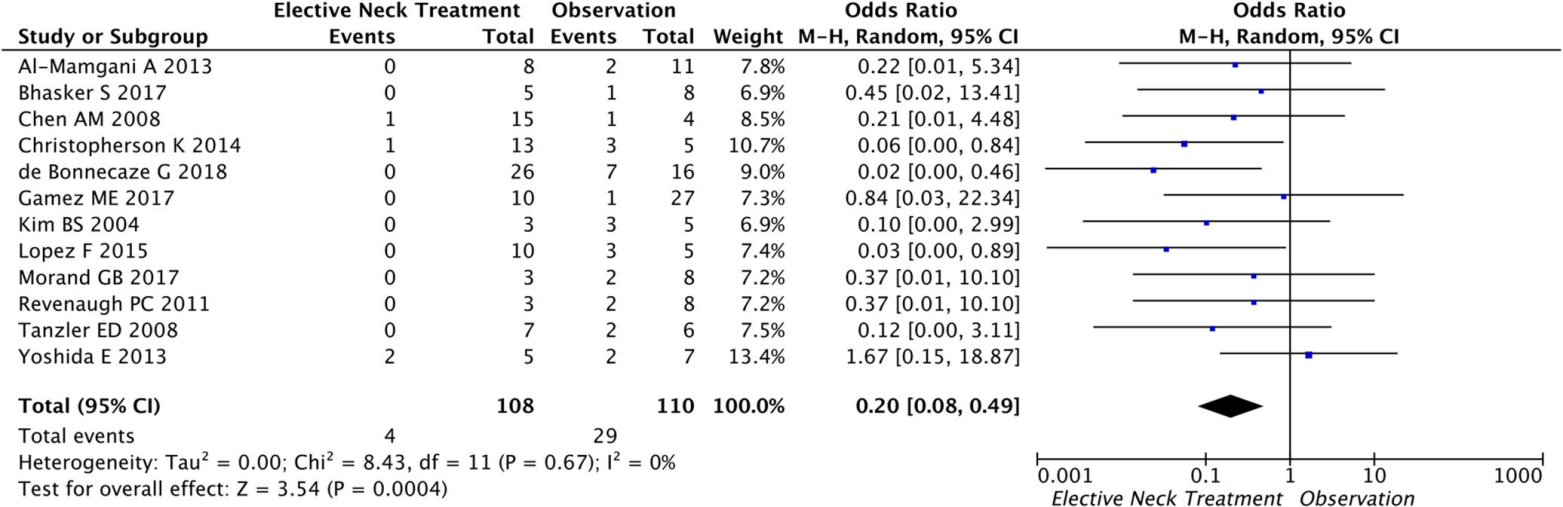
Meta‐analysis—elective neck treatment and locoregional control [Color figure can be viewed at http://wileyonlinelibrary.com]

Next we performed a meta‐analysis assessing regional (neck) relapse after elective neck treatment compared to observation in clinically N0 necks. Due to an insignificant heterogeneity (*I* ^2^ = 0) among included studies, the fixed‐form model was applied for analysis. Data of 218 patients was available for analysis, of whom 108 (49.5%) underwent elective neck treatment and 110 (50.5%) did not. Regional relapses occurred in 3.7% (4/108) patients with elective neck treatment compared to 26.4% (29/110) without. Accordingly, ORs for regional relapse after elective neck treatment ranged from 0.02 to 1.67, respectively. However, the pooled OR was 0.20 (95% CI 0.08‐0.49; *P* = .0004), indicating an 80% lower odd for regional nodal relapse in patients with elective neck treatment compared to those without (Figure 3).

### 3.5 Quality of studies

The risk of bias of included works has been assessed in eight categories using the ACROBAT‐NRSI tool recommended by the Cochrane group (Table 3) and an overall score was calculated indicating the quality of each analyzed study. Altogether, one study was quantified with a low risk, six studies with a moderate risk, and five studies were categorized with a serious risk of bias. In particular, 6 (50.0%) and 4 (33.3%) studies were rated with a high risk of bias in the categories “match with prognostic variables” and “Co‐intervention between groups” (Table 3).

**Table 3:** Quality of included studies (ACROBAT NRSI Version 10)

	Assessment of BIAS								
Study	Selection of population	Assessment of exposure	Outcome of interest	Match with prognostic variables	Assessment of prognostic factors	Assessment of outcome	Adequate follow up	Co‐intervention between groups	Overall score
Al‐Mamgani et al	Low risk	Low risk	Low risk	High risk	Low risk	Low risk	Low risk	High risk	High risk
Bhasker et al	Low risk	Low risk	Low risk	High risk	Low risk	Low risk	Low risk	Medium risk	High risk
Chen et al	Low risk	Low risk	Low risk	Medium risk	Low risk	Low risk	Low risk	Medium risk	Medium risk
Christopherson et al	Low risk	Low risk	Low risk	Low risk	Low risk	Low risk	Low risk	Low risk	Low risk
de Bonnecaze et al	Low risk	Low risk	Low risk	High risk	Low risk	Low risk	High risk	High risk	High risk
Gamez et al	Low risk	Medium	Low risk	High risk	Low risk	Low risk	Low risk	High risk	High risk
Kim et al	Medium risk	Low risk	Low risk	High risk	Low risk	Low risk	Low risk	Medium risk	Medium risk
Lopez et al	Low risk	Low risk	Low risk	Medium risk	Low risk	Low risk	Low risk	Low risk	Medium risk
Morand et al	Low risk	Low risk	Low risk	Low risk	Low risk	Low risk	Low risk	Medium risk	Medium risk
Revenaugh et al	Low risk	Low risk	Low risk	High risk	Low risk	Low risk	Low risk	High risk	High risk
Tanzler et al	Low risk	Low risk	Low risk	Low risk	Low risk	Low risk	Low risk	Medium risk	Medium risk
Yoshida et al	Low risk	Low risk	Low risk	Low risk	Low risk	Low risk	Low risk	Medium risk	Medium risk

Abbreviation: ACROBAT NRSI, A Cochrane Risk of Bias Assessment Tool for Non‐Randomized Studies of Interventions.

[Correction added on 3rd April 2020, after first online publication: “Morand and Irinakis” has been changed to “Morand et al” in column 1.]

## 4 DISCUSSION

SNUCs are rare sinonasal malignancies with an estimated incidence rate of 0.02 per 100 000 person‐years^11^ that typically occur within the fifth decade, and more likely in men than women.^24^, ^29^, ^30^ Owing to the rarity of the disease, the aggressive tumor behavior and poor outcome, defined management protocols and recommendations are lacking.

For primary disease control, multimodality treatment regimens combining surgery with radiotherapy and/or chemotherapy, has been widely acknowledged based on published series.^31^, ^32^ Our data are in accordance to literature, showing that trimodality therapy had the best LRC (63.9% ± 20.5%) followed by bimodality treatment regimes (49.2% ± 21.5%) and surgery alone (31.3% ± 9.8%). Importantly, locoregional failures have been associated with worse prognosis. Despite all these existing data, prophylactic neck treatment in the absence of clinical or radiological findings has been a subject of controversy,^15^, ^16^, ^33^, ^34^ with no clear recommendations regarding elective neck treatment.

Within this meta‐analysis we evaluated neck treatment regimes of 12 different works regarding to regional failure in 218 patients with clinically negative (cN0) disease. Among them, 108 patients have been treated with elective neck treatment (either irradiation or dissection) and only 4 (3.7%) patients had neck failures. Contrary to that, 110 patients did not receive neck treatment and 29 (26%) have resulted in regional recurrences. Altogether, elective neck treatment was associated with an 80% decreased risk for regional recurrence.

Previous working groups have utilized neck irradiation as part of treatment protocol with an anticipated high risk of regional failure.^19^, ^20^, ^27^, ^28^, ^35^ Principally, either END or elective neck irradiation (ENI) could be applied to address regional control.

Due to limited publications and small number of patients for series published over years, Mirghani et al^31^ has raised the concern over under diagnosis of nodal involvement in SNUC due to unavailability of advanced imaging modalities. Delayed presentation of sinonasal cancers with extension to adjacent sites such as nasopharynx and oral cavity has also been thought about as one of the factors for regional metastasis due to rich lymphatic network. Kim et al^23^ have found such relation to exist as the risk of regional recurrences increases with oral cavity involvement. Contradictory to that, Le et al^17^ and Paulino et al^18^ have failed to identify this association. Another issue identified with previous literature was clear demarcation between local and regional failures as a result of which the exact risk of nodal failures could not be interpreted.^17^, ^36^

A combined rate of initial nodal involvement and subsequent nodal recurrence of ≥15% has typically been applied as a threshold for elective neck treatment.^37^ Accordingly, a mean incidence of positive neck nodes of 14.1% accompanied by a risk of regional failure of 26.4% in cN0 patients without elective neck treatment justifies elective neck treatment.

Noteworthy, the extent of elective neck treatment, balancing potential harm as well as benefit, is not well described in the literature. Although comprehensive bilateral elective neck treatment of level I‐V would likely lead to the lowest number of regional recurrences, significant side‐effects of bilateral ND (eg, tracheostomy) or irradiation (eg, dysphagia) may lead to refusal and rejection of therapy, resulting in poor outcome again. Therefore, an appropriate extent of elective neck treatment is crucial. Ahn and coworkers^37^ noticed that nodal metastasis were associated with locality of SNUCs in sinonasal region, and that the most common nodal basin to harbor metastasis from nasal and ethmoid area was level II‐III, while nonnasal and nonethmoidal subsites drained into bilateral level I‐III. Similarly, Al‐Mamghani et al^19^ recommended ENI (45‐50 Gray) for level I‐III to reduce the risk of regional failure. In accordance to literature and our data, elective neck treatment of level I‐III is recommendable for patients with SNUCs.

We further recommend choosing END over irradiation to avoid short as well as long term side effects of radiotherapy, such as xerostomia, hair loss, tissue fibrosis, osteoradionecrosis and dental caries.^38^ Neck dissection, in addition to reducing the risk of regional failure, also provides the histological tissue diagnosis for accurate staging and identifying adverse features to further apply adjuvant radiation or chemotherapy.^39^ Elective neck treatment with radiation rather than surgery usually restricts the options of re‐irradiation in case of recurrence. Although salvage neck dissection following radiation treatment may exist as an option, such surgery in a previously irradiated bed may have deleterious side effects and complications in the form of delayed healing, wound infection or break down and vulnerability of major vessels to life threatening rupture.^40^

Our study has its share of limitations in the form of certain bias in some of the reviewed articles that has been compensated in the meta‐analysis. The published series are retrospective in nature and have no clear recommendations over which nodal levels to be addressed. The benefit of ipsilateral or bilateral neck treatment has still remained a subject of debate to be addressed in future studies. Importantly, 96.1% of our cases were T3 and T4 SNUCs, and therefore, our data endorse elective neck treatment particularly in advanced stage SNUCs.

## 5 CONCLUSION

Elective neck treatment is associated with significantly better regional control and less nodal relapses especially in patients with T3 and T4 SNUCs, and therefore, we recommended at least ipsilateral level I‐III treatment. Whether or not, midline tumors may benefit from bilateral elective neck treatment, still needs to be further elucidated.

## CONFLICT OF INTEREST

The authors declare no conflicts of interest.
